# Clinical Remarks upon Surgical Cases in the Buffalo General Hospital—Congenital Cataract—Perineal Section for Relief of Impassable Stricture

**Published:** 1869-04

**Authors:** J. F. Miner


ART. III.—*Clinical Remarks upon Surgical Cases in the Buffalo General Hospital—Congenital Cataract—Perineal Section for relief of impassable Stricture.* BY J. F. MINER, M. D.

*Congenital Cataract.*—The young lad which we present before you this morning has cataract in both eyes, which was observed to be present a few months after birth. Such disease when present at, or appearing soon after birth, is called congenital cataract—is called so if it appears at a later period, even after the age of puberty. Whether existing in infancy or afterward, the appearances are the same, and are quite different from those cataracts which appear in elderly people; the grayish or blush-white appearance of the opacity is quite characteristic. Sometimes the opacity occupies only the central portion of the lens, but it almost always extends until the whole is opaque. You will observe the constant rolling of the eyes—oscillation as it is called; this is commonly present where blindness has existed from birth, the patient never having learned to control the motions of the eyes, from having been unable to fix them upon objects.

Hereditary predisposition is said to be the frequent cause of congenital cataract; certain it is, that the disease does sometimes affect several members of the same family. Beyond this I am unable to give you any cause for its appearance, at birth; when it appears later, more obvious causes might be in operation. The prognosis is generally very favorable, and though the case before you has been operated upon, as I understand twice, thus far unsuccessfully, still I have little doubt it can be removed. Thus far it appears the opaque lens has resisted the solvent or absorbent action of the aqueous humor; but it is highly probable that if the capsule is thoroughly divided, and the lens broken up, that the opacity will disappear. It is true it may resist a first and second operation, but we will give the lens a complete division, freely rupturing the capsule, and it must eventually conform to the almost universal rule.*

Congenital cataract comes under the general head of soft cataract, and therefore may be removed without extraction. It is so soft that when the capsule is ruptured freely, nothing further need be attempted; it will be removed by the natural absorbent action of the aqueous humor, which is now freely admitted to the lens. I have previously described to you this operation and I have no occasion to repeat. The pupil should be dilated. The patient, usually a child, should be anæsthetized so as to ensure greater immobility, and the globe may be controlled by seizing the conjunctiva with fine forceps. I have introduced the needle, as you observe, through the sclerotic, about a line from the border of the cornea, and thus am able to divide capsule and lens most thoroughly, as I am determined to give it a fair chance to be removed; but the needle may also in the operation for congenital cataract, be introduced through the cornea, and the same object attained by slight modification of the procedure. The time requisite for absorption varies in different cases, depending upon the freedom of the division of lens and capsule, softness of the lens and vigor of the patient. A few weeks are generally quite sufficient in congenital cataract for complete absorption; the lens in such cases is softer than in cataract in advanced life except when these latter have resulted from injury.

*Perineal Section for Stricture.*—Our second patient, Mr. D., is about thirty-two years old; otherwise than the stricture is very healthy. The history which he gives of his case is, that when quite young he contracted a severe gonorrhœa, which resulted in stricture of the urethra. He soon noticed his condition, but neglected attending to it. He says that for the past few years he has been constantly engaged in fruitless and painful efforts to void his urine, and that life in his present situation is not to be endured. Within a year or two various physicians have had him in charge, but no relief has been obtained. Much time has been spent in efforts to pass instruments into the bladder, but no one

* Two months after operation patient returns with perfect result in the eye twice before operated upon, and the lens nearly absorbed in the other eye. Amount of vision not accurately tested for want of opportunity. Oscillation still present, which must greatly interfere with near vision.

has ever yet succeeded. For the last few months he has been under the care of Dr. Phelps of this city, who has made persevering effort to overcome the stricture by dilatation. He has done this so faithfully and judiciously that I accept his experience as my own, and proceed to attempt one of the most difficult and uncertain operations in surgery. I have already accomplished it in three instances in my life, each time without so much delay and embarrassment as I expected, but always with the sincere hope that I might never have occasion to repeat it. Perineal section, for relief of stricture, is only necessary in extreme cases; the occasion for making it is very infrequent since so many means are now at command to avoid it, but there are cases which resist all other modes of procedure, and to these few cases the operation is applicable.

The incisions are made similar to cutting for stone, but when you reach your staff, which has been introduced as far as the urethra is pervious, you may have no further guide, all track and trace of a urethra are absent, and you are to seek unguided the urethra upon the other side of the stricture. It would often be impossible to do this, but for the fact that the constant effort in voiding urine has had the effect to dilate the urethra back of the stricture, and thus make it more obvious to the touch and more likely to be opened into. We have succeeded after much effort, delay and searching to find, I was about to say, the urethra behind the stricture, but really have passed back into the bladder, without any guide at all, so that instruments are now passed easily through our perineal incision into the cavity of that viscus. Such an operation is one of the most uncertain and embarrassing which surgeons ever undertake, and withal is attended with dangers to the life of the patient so great that other, safer and more simple plans, should be thoroughly tried before resorting to it. I shall watch the progress of the case with deep interest and will report to you the result.* The after treatment will consist mainly in keeping a catheter in the urethra, changing it often to prevent deposit upon it, and allowing the parts to heal over the instrument, thus establishing a new and pervious urethra.

---

* At present writing, the patient is again at work as a laborer. Dr. Phelps, who has had care of him, informs me that the incision healed kindly and that urination is now free and painless.

